# Trajectory tracking control of parafoil systems via soft actor-critic with benchmark task set generation

**DOI:** 10.1038/s41598-026-54074-2

**Published:** 2026-05-22

**Authors:** Yufeng Fan, Lulu Ning, Xiang Wu, Yu Yan, Caixia Su, Pengju Wang, Yongfeng Cao

**Affiliations:** 1 School of Mathematical Sciences, Guizhou Normal University, Guiyang, 550025 China; 2 College of Bioresources Chemical and Materials Engineering, Shaanxi University of Science and Technology, Xi’an, 710016 China; 3 School of Big Data and Computer Science, Guizhou Normal University, Guiyang, 550025 China; 4 Finance Department, Guizhou University of Finance and Economics, Guiyang, 550025 China

**Keywords:** Parafoil system, 9-DOF, Trajectory tracking, Benchmark task set generation (BTSG), SAC, Engineering, Mathematics and computing

## Abstract

Parafoil systems are increasingly deployed in military and civilian missions that require high-precision trajectory tracking. However, conventional controllers based on linear strategies and limited decision information often fail to cope with task complexity, and existing reinforcement learning studies rarely consider the systematic construction of training task sets, thereby limiting generalization. To address these issues, this paper proposes a Benchmark Task Set Generation (BTSG) algorithm that builds high-quality task sets with diverse and balanced complexity for robust controller training and comprehensive evaluation, formulates the trajectory tracking problem as a Markov decision process with a 46-dimensional observation space and a dense reward structure to enrich decision-support information and improve learning efficiency, and develops a BTSG-SAC framework that integrates BTSG with Soft Actor-Critic (SAC) for controller training. Simulation results show that BTSG-SAC markedly outperforms PID and PPO controllers on the generated benchmark task set, reducing distance deviation by 50.7% and 33.2% under noise-free conditions; under sensor noise, BTSG-SAC attains a 98% success rate, whereas PPO reaches 77% and PID fails to achieve successful tracking. Overall, this paper extends the application of reinforcement learning to parafoil control and offers generalizable methodologies for future autonomous control research.

## Introduction

Parafoil systems are efficient airdrop devices that typically consist of a canopy, a payload, and connecting suspension lines. Owing to their inherent flight controllability and high payload capacity, they have been widely employed in both military and civilian sectors and are increasingly considered for applications such as payload recovery^1^ and space-related missions^2–4^. As application scenarios continue to expand, parafoil systems are expected to perform more complex missions, which imposes stricter requirements on trajectory tracking accuracy. However, their dynamics are complex, nonlinear, and strongly coupled, and the systems remain highly sensitive to wind disturbances and trajectory complexity, making accurate trajectory tracking particularly challenging.

Given constraints related to cost, safety, and experimental efficiency, parafoil control studies are commonly carried out on simulation platforms for controller design and validation^5,6^. In such platforms, an accurate dynamic model that faithfully reproduces the system’s flight behavior is essential. Existing work has proposed dynamic models with between three and nine degrees of freedom (DOF)^7–12^. Higher-order models capture the system’s dynamics more comprehensively but at the cost of increased computational complexity. In a 6-DOF formulation, the canopy and payload are treated as a single rigid body that approximates the overall motion of the parafoil system and is widely adopted in trajectory tracking control research^13–15^. The 8-DOF model additionally represents the relative pitch and yaw motions between the canopy and payload, and has therefore become a popular choice in recent studies^16–18^. The 9-DOF model further extends the 8-DOF formulation by including relative roll motion between the canopy and payload. Cheng et al.^19^ employed this model to investigate the parafoil flare problem. Considering the structure of the parafoil prototype and the required simulation fidelity, the present study adopts a 9-DOF-based simulation platform for controller training and testing, with the aim of exploring high-precision trajectory tracking control algorithms that enhance the system’s performance and robustness.

A substantial body of research has investigated classical control strategies for trajectory tracking in parafoil systems. The most common approach is to design specific guidance laws and then design controllers that track the heading commands generated by these laws^20^. PID controllers, which are widely used in engineering^14^, aim to minimize the deviation between the heading of the parafoil system and the heading of the planned trajectory. To address the challenge of tuning PID parameters, they are often combined with algorithms such as particle swarm optimization. Tao et al.^21,22^ designed a heading angle guidance law incorporating lateral distance error and used active disturbance rejection control (ADRC) for planar trajectory tracking control. Simulation results have demonstrated that this approach outperforms traditional PID control. He et al.^23^ designed a guidance law integrating forward speed and heading angle, and improved ADRC by pre-smoothing the sideslip angle to enhance the stability of the system during path tracking. This control strategy was validated in a simulation environment. Tao et al.^24^ developed a heading angle guidance law and adopted a Linear Active Disturbance Rejection Control (LADRC) strategy with easier parameter tuning. This design allows tracking errors in parafoil systems to be corrected promptly. Zheng et al.^5,16,18^ used LADRC to track the yaw angle and introduced a deep reinforcement learning-based adaptive parameter tuning algorithm, enhancing the parafoil system’s disturbance rejection capability. Sun et al.^6^ employed a controller combining the extended state observer (ESO) and sliding mode control (SMC) for trajectory tracking, validated through hardware-in-loop simulation experiments. Li et al.^13^ designed a guidance law based on a combination of position error and velocity error, and also used SMC strategy for tracking. However, traditional controllers (e.g., PID, ADRC, and LADRC) are constrained by linear or weakly nonlinear control strategies, making it challenging to achieve accurate trajectory tracking in complex parafoil systems. Moreover, these controllers rely on limited information, such as heading error, time-varying heading error, and wind field information, which restricts their effectiveness in handling the complex changes of wind fields and planned trajectories during flight tasks. Compared to traditional control strategies, the deep reinforcement learning-based method proposed in this study benefits from the neural network’s superior nonlinear modeling capability and the designed 46-dimensional observation state that provides rich decision-making information.

Deep reinforcement learning (DRL) is an active area of research in artificial intelligence, enabling agents to learn strategies that maximize cumulative rewards through interaction with the environment^25^. The success of DRL in the gaming domain has encouraged its application to a variety of real-world decision-making and control problems^26,27^. AlMahamid and Grolinger^28,29^ proposed Agile DQN, an attention-enhanced DQN method for autonomous UAV obstacle avoidance in complex environments. Liu et al.^30^ proposed a reconfiguration control method that combines LADRC with the TD3 algorithm to enhance the adaptability and robustness of aircraft anti-skid braking systems under actuator faults and external disturbances. Goddard et al.^31^ proposed a method that integrates reinforcement learning with primitive-based game tree search to autonomously generate and evaluate motion primitives, enhancing planning efficiency and adversarial performance in continuous state and action spaces. Soleimani et al.^32^ combined multilayer neural networks with continual learning in an actor-critic framework for safe trajectory tracking of quadrotor formations. These works, however, target fixed-wing or rotary-wing platforms with full actuation; DRL-based trajectory tracking for underactuated parafoil systems, whose dynamics are more nonlinear and tightly coupled, has received comparatively little attention.

In recent years, its use in the autonomous control of parafoil systems has gradually increased. Yu et al.^33^ proposed an improved TD3 method for trajectory planning, enhancing real-time performance and landing accuracy. To address the challenges of LADRC parameter tuning, Zheng et al. applied deep reinforcement learning algorithms, including DQN^18^, DDPG^5^, and SAC^16^, to train policy networks as independent modules for parameter-adaptation, thereby enhancing trajectory tracking performance. However, in these methods, the policy networks receive limited information, which is mainly based on the input data used by traditional controllers, restricting the agent’s decision-making potential. Wei et al.^34^ used the Proximal Policy Optimization (PPO) algorithm to train a parafoil precision landing controller in a wind-disturbance environment. The controller receives state information such as the parafoil’s current position, attitude, velocity, and angular velocity, directly outputting control actions for accurate landing. Park^35,36^ trained a parafoil hazard avoidance landing controller using the SAC algorithm, where the controller takes a nadir-pointing grayscale image as input and outputs control actions for landing. However, these studies mainly focused on the landing phase and with limited attention paid to the trajectory tracking problem. He et al.^37^ proposed a simulation training system based on the PPO algorithm for training a trajectory tracking controller, which is the closest related work to this study so far. A common limitation across these studies is that training tasks are either hand-crafted or randomly sampled, with no systematic procedure to control the diversity and balance of the task set—an issue that can affect both controller generalization and the reliability of performance evaluation. To overcome this limitation, this study proposes a Benchmark Task Set Generation (BTSG) algorithm that systematically constructs benchmark task sets for robust controller training and reliable performance evaluation.

This study makes the following contributions:

- Motivated by the gap in task set construction within existing DRL-based studies, this paper introduces a Benchmark Task Set Generation (BTSG) algorithm that categorizes tasks according to wind field conditions and planned trajectory complexity. The proposed algorithm generates benchmark task sets with diverse and balanced complexity distributions, supporting robust controller training and comprehensive performance evaluation. In addition, its systematic design provides a reusable framework that can facilitate future research.
- To overcome the limitations of traditional controllers, this study formulates the parafoil trajectory tracking problem as an MDP with a 46-dimensional observation space and a dense reward function that facilitate efficient training and stable improvement. This formulation offers a generalizable methodology for applying reinforcement learning to trajectory tracking in underactuated aerial systems.
- A BTSG-SAC framework is proposed by incorporating BTSG into SAC for controller training. Extensive simulations demonstrate that BTSG-SAC controller significantly outperforms both the widely-used PID controller and PPO-based controller across diverse tasks under noise-free and noisy conditions.

## A brief introduction to the 9-DOF parafoil model

### Coordinate systems and assumptions

**Figure 1:**
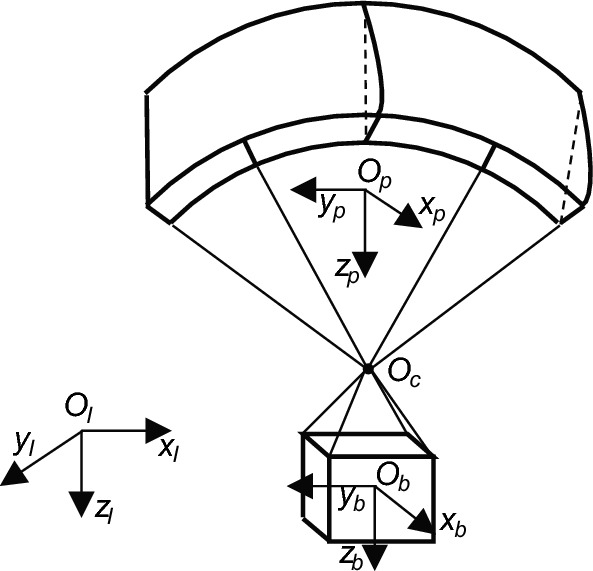
Schematic diagram of parafoil system.

As shown in Figure 1, the parafoil system primarily consists of a canopy and a payload, which are connected by suspension lines through a spherical joint that transmits force but not torque. The center of mass of the canopy is denoted as $O_p$, while the center of mass of the payload is denoted as $O_b$. The center of the spherical joint, $O_c$, serves as the formal connection point between the canopy and the payload.

To facilitate the modeling of the parafoil system, three coordinate systems are established, along with some basic assumptions: The ground coordinate system $O_I x_I y_I z_I$, the canopy coordinate system $O_p x_p y_p z_p$, the payload coordinate system $O_b x_b y_b z_b$. The assumptions are as follows: **(1)** The canopy maintains a fixed shape when fully inflated, and its center of mass overlaps with the center of aerodynamic pressure; **(2)** The suspension lines have a fixed length and form a single rigid body with the canopy; **(3)** The payload is treated as an independent rigid body, with only its aerodynamic drag being considered.

The transformation matrices from the ground coordinate system to the payload and canopy coordinate systems are as follows:

1 $$\begin{aligned} \boldsymbol{T}_b = \begin{bmatrix} c_{\theta _b} c_{\varphi _b} & c_{\theta _b} s_{\varphi _b} & -s_{\theta _b} \\ s_{\phi _b} s_{\theta _b} c_{\varphi _b} - c_{\phi _b} s_{\varphi _b} & s_{\phi _b} s_{\theta _b} s_{\varphi _b} + c_{\phi _b} c_{\varphi _b} & s_{\phi _b} c_{\theta _b} \\ c_{\phi _b} s_{\theta _b} c_{\varphi _b} + s_{\phi _b} s_{\varphi _b} & c_{\phi _b} s_{\theta _b} s_{\varphi _b} - s_{\phi _b} c_{\varphi _b} & c_{\phi _b} c_{\theta _b} \end{bmatrix} \end{aligned}$$

2 $$\begin{aligned} \boldsymbol{T}_p = \begin{bmatrix} c_{\theta _p} c_{\varphi _p} & c_{\theta _p} s_{\varphi _p} & -s_{\theta _p} \\ s_{\phi _p} s_{\theta _p} c_{\varphi _p} - c_{\phi _p} s_{\varphi _p} & s_{\phi _p} s_{\theta _p} s_{\varphi _p} + c_{\phi _p} c_{\varphi _p} & s_{\phi _p} c_{\theta _p} \\ c_{\phi _p} s_{\theta _p} c_{\varphi _p} + s_{\phi _p} s_{\varphi _p} & c_{\phi _p} s_{\theta _p} s_{\varphi _p} - s_{\phi _p} c_{\varphi _p} & c_{\phi _p} c_{\theta _p} \end{bmatrix} \end{aligned}$$

Where, $c_* = \cos (*)$, $s_* = \sin (*)$, $$\boldsymbol{W}_b = \begin{bmatrix} \varphi _b&\theta _b&\phi _b \end{bmatrix}^T$$ represents the Euler angles of the payload, and $$\boldsymbol{W}_p = \begin{bmatrix} \varphi _p&\theta _p&\phi _p \end{bmatrix}^T$$ represents the Euler angles of the canopy (where $\varphi$ is the roll angle, $\theta$ is the pitch angle, and $\phi$ is the yaw angle).

### The nine-DOF dynamic model of the parafoil system

The motion model of the parafoil system can be simplified to a three-degree-of-freedom translational motion at the connection point $O_c$, along with three-degree-of-freedom rotational motions of the canopy and payload around their respective centers of mass, $O_p$ and $O_b$. Based on this, a nine-degree-of-freedom (9-DOF) dynamic model of the parafoil system is developed, which incorporates the following kinematic and dynamic equations.

The kinematic equations are as follows:

3 $$\begin{aligned} \boldsymbol{\dot{X}_c} = \boldsymbol{V_c} \end{aligned}$$

4 $$\begin{aligned} \begin{bmatrix} \dot{\varphi }_b \\ \dot{\theta }_b \\ \dot{\psi }_b \end{bmatrix} = \begin{bmatrix} 1 & s_{\phi _b} t_{\theta _b} & c_{\phi _b} t_{\theta _b} \\ 0 & c_{\phi _b} & -s_{\phi _b} \\ 0 & s_{\phi _b}/c_{\theta _b} & c_{\phi _b}/c_{\theta _b} \end{bmatrix} \begin{bmatrix} p_b \\ q_b \\ r_b \end{bmatrix} \end{aligned}$$

5 $$\begin{aligned} \begin{bmatrix} \dot{\varphi }_p \\ \dot{\theta }_p \\ \dot{\psi }_p \end{bmatrix} = \begin{bmatrix} 1 & s_{\phi _p} t_{\theta _p} & c_{\phi _p} t_{\theta _p} \\ 0 & c_{\phi _p} & -s_{\phi _p} \\ 0 & s_{\phi _p}/c_{\theta _p} & c_{\phi _p}/c_{\theta _p} \end{bmatrix} \begin{bmatrix} p_p \\ q_p \\ r_p \end{bmatrix} \end{aligned}$$

The dynamic equations are as follows:

6 $$\begin{aligned} \begin{bmatrix} \boldsymbol{-M_b \tilde{R}_{cb}} & 0 & \boldsymbol{ M_b T_b} & \boldsymbol{T_b} \\ 0 & \boldsymbol{\tilde{R}_{cp}} & \boldsymbol{(M_p + M_a) T_p }& \boldsymbol{-T_p} \\ \boldsymbol{I_b} & 0 & 0 & \boldsymbol{-\tilde{R}_{cb} T_b} \\ 0 & \boldsymbol{I_p + I_a} & 0 & \boldsymbol{\tilde{R}_{cp} T_p} \end{bmatrix} \begin{bmatrix} \dot{\boldsymbol{\omega _b}} \\ \dot{\boldsymbol{\omega _p}} \\ \dot{\boldsymbol{V_c}} \\ \boldsymbol{F_c} \end{bmatrix} = \begin{bmatrix} \boldsymbol{B_1} \\ \boldsymbol{B_2} \\ \boldsymbol{B_3} \\ \boldsymbol{B_4} \end{bmatrix} \end{aligned}$$

The first-order ordinary differential equations defined by Eqs. 3–6 constitute the nine-degree-of-freedom (9-DOF) model of the parafoil system, and the detailed descriptions of these equations are provided in Appendix A. This model defines the system state variables as $$\boldsymbol{x} = \begin{bmatrix} \boldsymbol{X_c}, \boldsymbol{W_b}, \boldsymbol{W_p}, \boldsymbol{V_c}, \boldsymbol{\omega _b}, \boldsymbol{\omega _p}, \boldsymbol{F_c} \end{bmatrix}$$ consisting of 21 state components. Where $\boldsymbol{X_c}$, $\boldsymbol{V_c}$, and $\boldsymbol{F_c}$ denote, respectively, the position, velocity, and constraint force at the connection point $O_c$; $\boldsymbol{W_b}$ and $\boldsymbol{\omega _b}$ denote the Euler angles and the corresponding angular rates of the payload; and $\boldsymbol{W_p}$ and $\boldsymbol{\omega _p}$ denote the Euler angles and the corresponding angular rates of the canopy. By solving these equations using the fourth-order Runge–Kutta method, the real-time state of the parafoil system can be computed.

The parafoil is steered by pulling down the left or right brake line to deflect the trailing edge of the canopy; the resulting deflections $\delta _l$ and $\delta _r$ are the physical control inputs^17^. Their symmetric component $\delta _s = \min (\delta _l, \delta _r)$ enters the lift and drag coefficients, altering the canopy aerodynamic force $\boldsymbol{F_{ap}}$ in the $\boldsymbol{B_2}$ term of Eq. 6, while the asymmetric component $\delta _a = \delta _l - \delta _r$ enters the roll and yaw moment coefficients, altering the canopy aerodynamic moment $\boldsymbol{M_p^A}$ in $\boldsymbol{B_4}$; $\delta _a$ is the quantity that actually governs heading control. Full expressions for $\boldsymbol{F_{ap}}$ and $\boldsymbol{M_p^A}$ as functions of $\delta _s$ and $\delta _a$ can be found in^17^.

**Figure 2:**
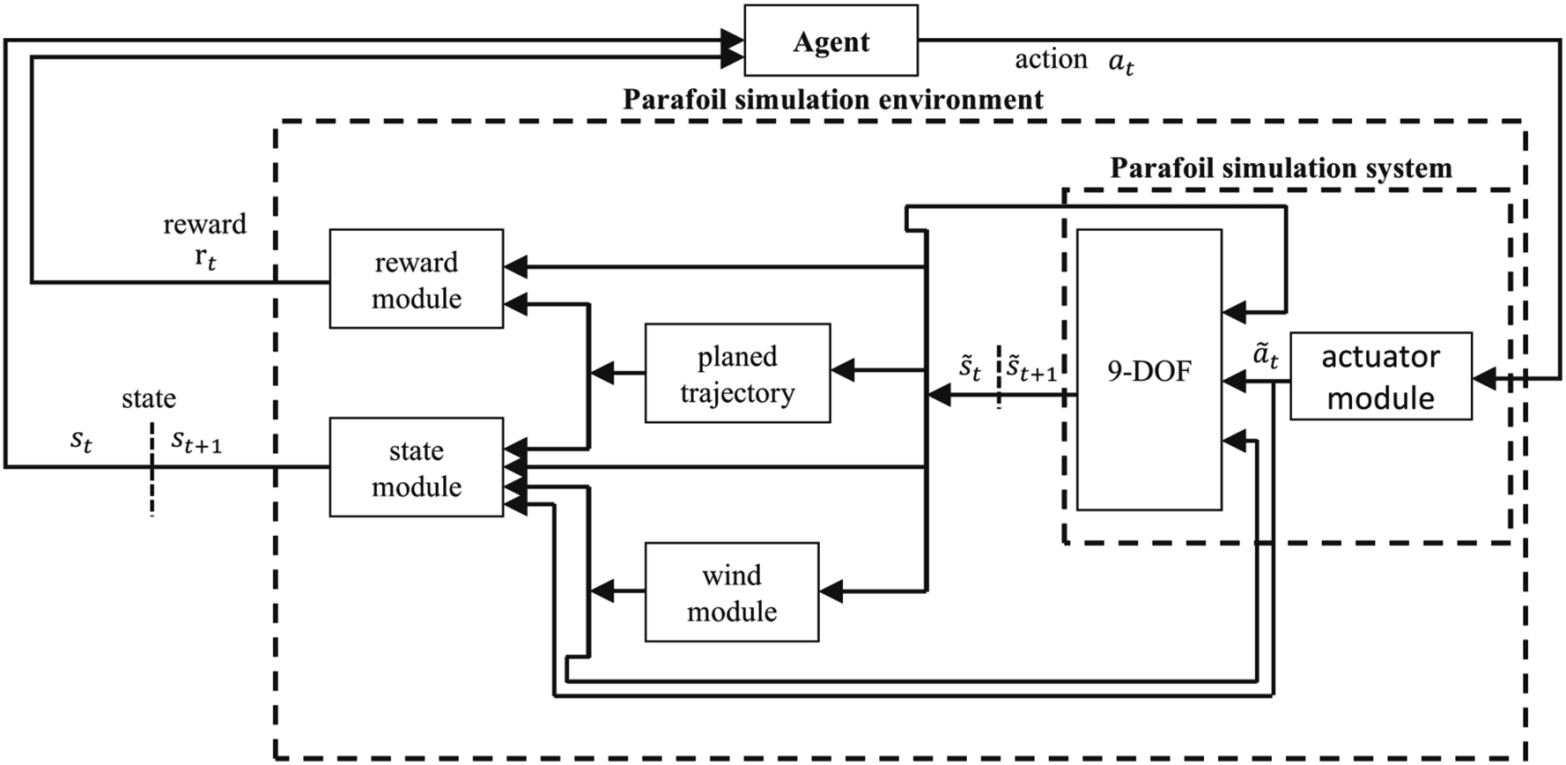
Schematic diagram of the interaction between Agent and parafoil simulation environment.

Based on the 9-DOF model, this study incorporates an actuator model^37^ to simulate the effects of the left and right motors pulling the control ropes, thereby constructing a parafoil simulation system, as illustrated in Figure 2. The simulation system is designed with an airspeed of 10.45 m/s and a glide ratio of 3.00, which translates to a horizontal velocity of 9.92 m/s and a vertical velocity of 3.30 m/s under no-wind conditions. All subsequent experiments are conducted using this simulation system.

## Benchmark task set generation algorithm

Constructing high-quality benchmark task sets is crucial to the practical application of reinforcement learning methods. Such task sets should provide sufficient diversity and appropriate balance to ensure that the training data is both comprehensive and representative while also meeting the scale requirements for model training. In conventional flight tasks (excluding extreme weather conditions such as rain or snow), the complexity of parafoil trajectory tracking is primarily influenced by wind field complexity and planned trajectory complexity. This section first presents a wind field modeling algorithm with tunable complexity settings and then defines and analyzes the complexity of planned trajectories. On this basis, we propose a Benchmark Task Set Generation (BTSG) algorithm that efficiently generates task sets under varying requirements. With BTSG, three task sets are constructed in this study. The first is a high-quality benchmark set with broad complexity coverage and balanced distributions for controller training and evaluation. The second and third are simple and complex sets, respectively, designed for comparative experiments with reference to the complexity distribution of the benchmark task set.

### Wind field modeling

The wind field plays a critical role in parafoil trajectory tracking tasks. Wind speed, wind direction, and wind disturbances are key factors determining task complexity and form the core elements defining wind field complexity. To improve computational efficiency and practical applicability, this study proposes a simplified wind field modeling method. The complex wind field is decomposed into the superposition of three components: mean wind, gusts, and random wind. The method incorporates wind field complexity control during modeling while restricting the wind field range to the vicinity of the parafoil trajectory. Based on this, a wind field generation algorithm is proposed.

**Mean Wind**. Mean winds refer to the average wind speed at various altitudes over a specific time period. This paper models mean wind using a power-law model, which describes the relationship between wind speed and altitude as:

7 $$\begin{aligned} V_{mean}\left( z\right) = V_{ref}\left( \frac{z}{z_{ref}}\right) ^\alpha \end{aligned}$$

Where: $V_{mean}(z)$ represents the average wind speed at altitude *z* (in meters); $V_{ref}$ denotes the wind speed (in m/s) at the reference altitude $z_{ref}$, which is randomly sampled within the range $[V_{m\_min}, V_{m\_max}]$; $z_{ref}$ is the reference altitude, which is set to 70 m in this study; $\alpha$ is the wind shear exponent, representing the rate of change in wind speed with altitude, and is set to 0.25 in this study. The mean wind direction $\boldsymbol{d_{mean}}$ is a unit vector in the ground coordinate system, typically set to a constant value that can be adjusted as a hyperparameter if needed. Under the above settings, $V_{m\_min}$ and $V_{m\_max}$ are the hyperparameters for the mean wind speed, which are key factors influencing wind field complexity and are positively correlated with complexity level.

The combination of Eq. 7 with the mean wind direction yields the formula for the mean wind vector as a function of altitude:

8 $$\begin{aligned} \boldsymbol{W_{mean}}\left( z\right) = V_{mean}\left( z\right) \cdot \boldsymbol{d_{mean}} \end{aligned}$$

**Gust**. Gusts refer to the phenomenon that wind speed suddenly increases and then decreases over a short period (typically a few seconds or minutes). This paper adopts the classical NASA gust model, where the variation of wind speed with altitude can be expressed as Eq. 9. Where: $V_{gust}(z)$ represents the gust wind speed increment at altitude *z*; $V_{gust_{max}}$ denotes the maximum gust wind speed increment, which is randomly sampled within the specified range $[V_{g\_min}, V_{g\_max}]$; $z_{gust_{min}}$ and $z_{gust_{max}}$ are the minimum and maximum altitudes influenced by the gust, respectively; $\delta z$ represents the altitude range over which the gust wind speed varies, meaning the interval where the gust increases from 0 to the maximum value (or decreases from the maximum value to 0). The gust wind direction, $\boldsymbol{d_{gust}}$, is a unit vector in the ground coordinate system and is uniformly randomly sampled in three-dimensional space. Under these settings, $V_{g\_min}$ and $V_{g\_max}$ are the second major factors influencing wind field complexity and are positively correlated with the complexity level.

9 $$\begin{aligned} V_{gust}\left( z\right) = {\left\{ \begin{array}{ll} V_{gust_{max}} \frac{1}{2} \left( 1 - \cos \frac{\pi (z - z_{gust_{min}})}{\delta z} \right) , & z_{gust_{min}} \le z< z_{gust_{min}} + \delta z \\ V_{gust_{max}}, & z_{gust_{min}} + \delta z \le z \le z_{gust_{max}} - \delta z \\ V_{gust_{max}} \frac{1}{2} \left( 1 - \cos \frac{\pi (z_{gust_{max}} - z)}{\delta z} \right) , & z_{gust_{max}} - \delta z < z \le z_{gust_{max}} \\ 0, & \text {otherwise} \end{array}\right. } \end{aligned}$$

Combining Eq. 9 with the gust wind direction, the formula for the gust vector as a function of altitude is:

10 $$\begin{aligned} \boldsymbol{W_{gust}}\left( z\right) = V_{gust}\left( z\right) \cdot \boldsymbol{d_{gust}} \end{aligned}$$

**Random Wind**. Random wind refers to the turbulence commonly found in natural wind fields, characterized by random and unpredictable wind speed variations. This paper models random wind using a Gaussian random process, where the variation of wind speed with altitude is expressed as:

11 $$\begin{aligned} V_{random}\left( z\right) \sim \mathcal {N}\left( 0, \sigma ^2\right) \end{aligned}$$

Where: $V_{random}(z)$ represents the random wind speed increment at altitude *z*; $\mathcal {N}(0,\sigma ^2)$ denotes a Gaussian distribution with a mean of 0 and a variance of $\sigma ^2$; $\sigma$ reflects the fluctuation intensity of the random wind speed and is uniformly sampled within the hyperparameter-defined range $[\sigma _{r_{min}}, \sigma _{r_{max}}]$. The random wind direction, $\boldsymbol{d_{random}}$, is a unit vector in the ground coordinate system and is uniformly randomly sampled in three-dimensional space. Under these settings, $\sigma _{r_{min}}$ and $\sigma _{r_{max}}$ are the third major factors influencing wind field complexity and are positively correlated with it.

Combining Eq. 11 with the random wind direction yields the formula for the random wind vector as a function of altitude:

12 $$\begin{aligned} \boldsymbol{W_{random}}\left( z\right) = V_{random}\left( z\right) \cdot \boldsymbol{d_{random}}\left( z\right) \end{aligned}$$

By superimposing the above three wind vectors, the wind vector at any altitude *z* can be obtained as:

13 $$\begin{aligned} \boldsymbol{W}\left( z\right) = \boldsymbol{W_{mean}}\left( z\right) + \boldsymbol{W_{gust}}\left( z\right) + \boldsymbol{W_{random}}\left( z\right) \end{aligned}$$

**Algorithm 1:**
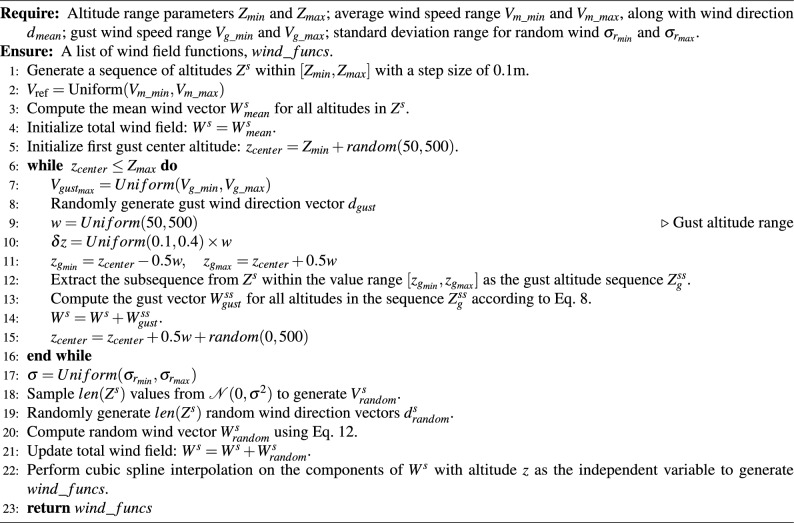
Wind Field Generation Algorithm.

Based on Eq. 13, this study proposes an algorithm for generating wind fields, with the pseudocode provided in Algorithm 1. By configuring the parameters $V_{m\_min}$, $V_{m\_max}$, $V_{g\_min}$, $V_{g\_max}$, $\sigma _{r_{min}}$, $\sigma _{r_{max}}$, the complexity of the wind field can be flexibly adjusted. The algorithm outputs a list of wind field functions, where the wind vector $\boldsymbol{W}(z)$ at a given altitude *z* ($z \in [Z_{min}, Z_{max}]$) can be obtained.

### Complexity of the planned trajectory and action sequence generation algorithm

The complexity of a planned trajectory is a critical factor influencing the overall difficulty of parafoil trajectory tracking tasks. This section begins by analyzing two basic types of planned trajectories and defining them as simple and complex. It then identifies the primary factors influencing their complexity. Subsequently, an action sequence generation algorithm with adjustable complexity is proposed. This algorithm is designed to be called by the BTSG algorithm described in Sect. Benchmark task set generation algorithm for generating planned trajectories.

Basic Motion Trajectory Types of the Parafoil System. Constrained by the control mechanisms of the parafoil system, its motion trajectories are classified into two basic types: linear trajectories and circular arc trajectories. Under no-wind conditions, when the pull-down amounts of both the left and right motors are zero (no pull-down operation), the parafoil follows a straight-line trajectory, with its horizontal projection appearing as a line segment, referred to as a linear trajectory. When the pull-down amount on only one side is non-zero and kept constant (single-side pull-down operation), the trajectory’s horizontal projection forms a circular arc with a fixed radius or curvature, known as a circular arc trajectory. Theoretically, when the pull-down amounts on both sides are non-zero, equal amounts produce a linear trajectory, while unequal amounts result in a circular arc trajectory. However, dual-side operations are typically limited to the landing phase. To ensure flight safety, dual-side operations are generally avoided during the trajectory tracking phase and traditional PID controllers also do not support such control actions. Therefore, this study does not further address such scenarios further. Under windy conditions, both basic trajectory types experience deviations due to wind disturbances. Based on the above analysis, any motion trajectory can be regarded as the concatenation of several basic trajectories connected end-to-end. In this study, a trajectory composed of heterogeneous basic segments is referred to as a hybrid trajectory. To ensure effective trajectory tracking, planned trajectories must adhere to these motion constraints. Therefore, any complete planned trajectory can be represented as a hybrid trajectory, in which the constituent basic trajectories are referred to as sub-trajectories.

Planned trajectories are categorized as simple or complex based on their trackability by controllers. Simple planned trajectories are composed of relatively long basic trajectories. Key features of simple trajectories include larger radii of circular arc sub-segments, which reduce turning difficulty; a higher proportion of linear sub-trajectories, which facilitate tracking; and longer sub-segments, which reduce transitional controls between segments, thereby improving tracking stability. Consequently, these trajectories are generally easier to track and exhibit lower overall complexity. In contrast, complex planned trajectories are composed of many short basic trajectories. Their key features include smaller radii of circular arc sub-trajectories, which increase turning difficulty; a lower proportion of linear sub-segments, which complicate tracking; and shorter sub-segments, which increase the required transitional control between segments, thereby compromising tracking stability. Consequently, these trajectories are typically more difficult to track and exhibit higher overall complexity. In summary, the complexity of planned trajectories is primarily influenced by the radii of circular arc trajectories, the proportion of linear trajectories, and the length of sub-segments, all of which exhibit a negative correlation with complexity.

Action Sequence Generation for Planned Trajectories. In model-based planned trajectory generation methods, planned trajectories are equivalent to action sequences. Each element in the action sequence is represented by an action vector pair $(a, \Delta t)$. Here, *a* represents the control action vector, which determines the radius of the circular arc sub-trajectory; $\Delta t$ represents the execution time of the action (in seconds), which determines the length of the sub-trajectory. Executing the action sequence in the parafoil simulation environment generates the corresponding planned trajectory. Action sequences with different complexities can generate planned trajectories with different complexities. The pseudocode for generating the action sequence is shown in Algorithm 2. The input parameters of the algorithm include $a_{\min }$, $a_{\max }$, $t_{\min }$, and $t_{\max }$, where the first two determine the radii of the circular arc sub-trajectories and the proportion of linear sub-trajectories, while the latter two determine the lengths of the sub-trajectories. By setting the parameters, the complexity of the planned trajectory can be roughly defined. The return value of the algorithm is the generated list of action sequences.

**Algorithm 2:**
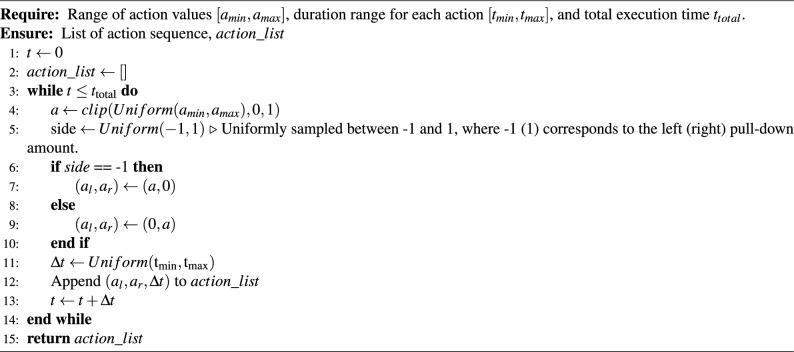
Action Sequence Generation Algorithm.

In Algorithm 2, the input parameters must satisfy the following constraints: $-1 \le a_{min} \le 1$, $0 \le a_{max} \le 1$, and $a_{min} \le a_{max}$. Particularly, when $a_{min} \le 0$, the truncation performed by the *clip()* function gives the action a certain probability of being 0, which corresponds to a linear trajectory. In this case, the value of $a_{min}$ determines the proportion of linear trajectories in the planned trajectory, which can be calculated as $\frac{-a_{min}}{a_{max} - a_{min}}$.

### Benchmark task set generation algorithm

Based on the above methods, this study introduces a Benchmark Task Set Generation (BTSG) algorithm. Each task is defined by a planned trajectory together with its corresponding wind field function. The complexity of the task set can be adjusted through a set of predefined parameters, allowing the algorithm to accommodate different experimental requirements. The main steps of the algorithm are as follows:

(1) Run Algorithm 1 to generate wind field functions;

(2) Run Algorithm 2 to generate action sequences;

(3) Use the wind field functions in the parafoil simulation environment to perform flight simulations based on the action sequences, while recording trajectory states at each time step;

(4) Save the trajectory state data and wind field functions as a flight task file.

To address the computational inefficiency caused by the parafoil simulation environment (due to complex computations and frequent control interactions), BTSG implements a parallel framework. The algorithm defines a parallel task function *parallel_task* that distributes tasks across multiple worker processes, significantly accelerating task set generation. The pseudocode for the algorithm is provided in Algorithm 3.

**Algorithm 3:**
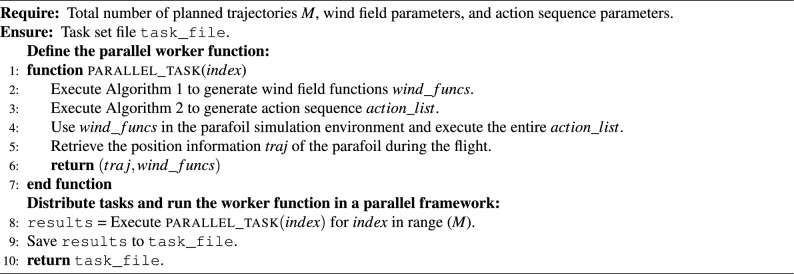
Benchmark Task Set Generation Algorithm.

Based on the proposed algorithm, this study builds three types of task sets. Each type consists of a training set and a separate evaluation set, giving six task sets in total, defined as follows:

**(1)** High-Quality Benchmark Task Set. This set includes diverse wind fields and planned trajectories. It serves both to train accurate controllers and to evaluate their overall performance. The wind speed ranges from [0, 8] m/s, the action value for generating trajectories ranges from [0, 0.6], and the duration of each action spans [30, 180] seconds (resulting in cumulative descent altitude of approximately [100, 600] meters). The proportion of linear sub-trajectories is 40%. The training set is referred to as HTT (High-Quality Benchmark Task Set for Training), while the evaluation set is referred to as HTE (High-Quality Benchmark Task Set for Evaluating).

**(2)** Simple Task Set. This task set comprises wind fields and planned trajectories with relatively low complexity and is primarily used for training and evaluating controllers under simple scenarios. The wind speed ranges from [0, 4.0] m/s, the action value for generating trajectories ranges from [0, 0.2], and the duration of each action ranges from [90, 180] seconds (resulting in cumulative descent altitude of approximately [300, 600] meters). The proportion of linear sub-trajectories is 50%. The corresponding training and evaluation sets are denoted by STT (Simple Task Set for Training) and STE (Simple Task Set for Evaluating), respectively

**(3)** Complex Task Set. This set includes diverse wind fields and complex planned trajectories. It is mainly used for training and evaluating controllers in complex scenarios. The wind speed ranges from [0, 8] m/s, the action value for generating trajectories ranges from [0, 0.6], and the duration of each action ranges from [30, 60] seconds (resulting in cumulative descent altitude of approximately [100, 200] meters). The proportion of linear sub-trajectories is 20%. The training set is referred to as CTT (Complex Task Set for Training), while the evaluation set is referred to as CTE (Complex Task Set for Evaluating). Compared to the high-quality task set, the complexity of the complex task set lies in its shorter sub-trajectories (requiring more transitional operations) and a lower proportion of linear sub-trajectories. The specific parameters for generating the above task sets are detailed in Appendix B.

## MDP formulation and SAC-based controller training

This section focuses on the SAC component of the BTSG-SAC framework. It first formulates the parafoil trajectory tracking control problem as an MDP and then presents the SAC4Para algorithm, a SAC-based controller training method, to solve the MDP.

### Formulating parafoil trajectory tracking control as an MDP

A Markov Decision Process (MDP) is an important abstract model for goal-oriented interactive learning problems, and it forms the core theoretical framework of reinforcement learning. MDP simplifies complex real-world interactions into the agent-environment interaction and abstracts this process into mathematical components, including state, action, reward function, and state transition function. This abstraction facilitates research and analysis. The agent generates an action based on the current state of the environment, and the environment responds to this action, transitioning to a new state and producing a corresponding reward. This interaction continues until the environment reaches a terminal state. The complete cycle, including state observation, decision execution, and state transition, is referred to as a time step. Typically, the first step in applying reinforcement learning to real-world problems is modeling the problem as an MDP, which involves clearly defining its components. Figure 2 illustrates the interaction process between the agent and the parafoil simulation environment. The parafoil simulation environment includes the parafoil simulation system, planned trajectory, wind field module, reward module, and state module. The following details the design of the state, action, reward function, and state transition function for modeling the parafoil trajectory tracking control problem.

**Table 1:** Details of the observation state sub-vectors.

Sub-vector Name	Symbol	Dimension
C Point Velocity	CV	3
Proximal Target Point Vector	PTV	2
Proximal Target Point Angle	PTA	1
Distal Target Point Vector	DTV	2
Distal Target Point Angle	DTA	1
Forward Trajectory Curvature	FTC	8
Forward Trajectory Heading Vector	FTV	24
Actuator Control Outputs	ACO	2
Wind Vector	WV	3

**State**. The state refers to the information observable by the agent from the parafoil simulation environment. The 21-dimensional nine-degree-of-freedom model constitutes the system state. Not all components of the system state, however, are equally accessible during actual flight. The planned trajectory is known a priori, and the control motor status is directly readable. C-point position and velocity are available through navigation receivers and accelerometers, while local wind conditions are recorded by pitot tube sensors. The canopy Euler angles and angular velocities, on the other hand, remain poorly observable given the impracticality of mounting sensors directly on the flexible canopy surface. Given these constraints, the observation state is constructed from quantities that are reliably measurable in flight: C-point position and velocity, the planned trajectory, control motor status, and local wind measurements. Through mathematical derivation of these quantities, a 46-dimensional state vector is formed, comprising 9 sub-vectors, each carrying a distinct physical interpretation. These sub-vectors are listed in Table 1; the corresponding physical definitions are detailed in Appendix C. This formulation supplies the agent with sufficient information for trajectory tracking decisions.

**Action**. The action is defined as a one-dimensional vector representing the control action output by the controller. Its value is normalized to be compatible with different types of parafoil systems. After normalization, the action value range is [-1, 1]. When the action value is negative, it indicates that the left side control rope is pulled down; when positive, it indicates that the right side control rope is pulled down. The absolute value of the action represents the target pull-down length of the control rope. A value of 0 indicates no pull-down on the control rope, while -1 and +1 denote the maximum permissible pull-down on the left and right sides, respectively. In the parafoil simulation system, the actuator model^37^ is responsible for interpreting and executing this action. The input signal of this model is a one-dimensional control action vector, and the output signal is the execution result of the left and right motors (a two-dimensional vector). The normalized control action defined here corresponds to the brake deflections $\delta _l$ and $\delta _r$ introduced in Sect. The nine-DOF dynamic model of the parafoil system, which are physically realized through the actuator model.

**Reward**. To address the challenges arising from the long duration and numerous interaction steps of the complete trajectory tracking task, this study defines the trajectory tracking problem as an episodic task. The maximum number of interaction steps per episode is set to S (where S = 100 and the interaction frequency is 1 Hz), and the maximum Euclidean distance between the parafoil simulation system and the planned trajectory at the same altitude is restricted to remain within the maximum safety distance, D (where D = 200 m). This setting helps reduce the training difficulty of the agent and simplifies the design of the reward function.

The goal of parafoil trajectory tracking control is to enable the system to closely follow the planned trajectory during flight through adjustments made by the controller. Based on this goal, a dense reward function combining heading angle deviation and horizontal distance deviation is designed. In addition, to prevent the agent from prematurely ending the task under dense rewards, the reward for each time step is weighted according to the actual number of interaction steps in that episode. The specific design of the reward function is as follows:

14 $$\begin{aligned} r_t = \mu (\alpha H_t + \beta D_t + \gamma C_t) \end{aligned}$$

where *t* represents the current interaction time step, $r_t$ is the current reward; $\alpha$, $\beta$, $\gamma$, and $\mu$ are the weight factors; $H_t$ is the heading angle deviation term, $D_t$ is the distance deviation term, and $C_t$ is the control smoothness term. In this study, $\alpha = 0.5$, $\beta = 0.4$, $\gamma = 0.1$, and $\mu = \frac{N}{S}$. Here, *N* represents the total number of actual interaction steps in the episode, and *S* denotes the maximum allowable number of interaction steps. The calculation methods for each reward sub-item are detailed in Appendix D.

In the reward function, $H_t$ penalizes the heading deviation via an exponential form $\exp (-A_t)$ that amplifies large errors, $D_t$ linearly penalizes horizontal distance up to the 200 m safety boundary, and $C_t$ penalizes consecutive-action differences to suppress actuator oscillations (see Appendix D for formulas). Both $H_t$ and $D_t$ are needed: distance minimization alone permits serpentine approach with wrong heading, while heading minimization alone does not ensure lateral convergence—only jointly driving both to zero keeps the parafoil on the planned trajectory. The weights $\alpha =0.5$, $\beta =0.4$, $\gamma =0.1$ allocate 90% of the penalty to tracking accuracy, empirically tuned for a precision-first objective. A dense per-step reward is used because episodes run up to $S=100$ steps, where a sparse terminal signal would provide insufficient learning feedback. The factor $\mu =N/S$ prevents the agent from favoring early termination of short episodes.

After training the controller with reinforcement learning algorithms and achieving convergence(In reinforcement learning, “convergence” generally refers to the agent’s performance—typically measured by cumulative reward—becoming stable during training. In this study, convergence is further required to imply that the agent has acquired an effective policy that achieves high cumulative rewards.), the agent can maximize the cumulative reward while controlling the parafoil system for trajectory tracking. Based on the reward function design in this study, maximizing the cumulative reward corresponds to simultaneously minimizing both horizontal distance and heading angle deviations, which means the parafoil system flies close to the planned trajectory—the ultimate goal of the trajectory tracking task.

**State Transition**. As shown in Figure 2, the process where the parafoil simulation environment changes from state $s_t$ to state $s_{t+1}$ under the action $a_t$ is called a state transition. The foundation of the state transition is the 9-DOF model. Based on the 21-dimensional system state $\tilde{s}$ generated by the 9-DOF model, and incorporating the planned trajectory and wind field model, the state module outputs the observation state for the next time step, while the reward module outputs the reward $r_t$ after executing action $a_t$.

### SAC-based controller training

This subsection presents SAC4Para, a SAC-based algorithm for training the parafoil trajectory tracking controller. The agent is trained through multiple episodes of interaction with the parafoil simulation environment, using benchmark task sets generated by the BTSG algorithm (Sect. Benchmark task set generation algorithm).

#### Soft actor-critic algorithm foundation

This study adopts an off-policy algorithm to train the parafoil controller, since agent–environment interaction dominates the training cost in our simulator and off-policy methods can reuse past transitions through a replay buffer^38^. PPO, as the algorithm used by He et al.^37^, is retained as an evaluation baseline (Sect. Performance evaluation). Among off-policy candidates, DDPG is known to be hyperparameter-sensitive and prone to training instability^38^. TD3 improves on DDPG via clipped double-Q learning, but in preliminary runs on our task SAC converged more reliably, likely because its maximum-entropy objective drives broader exploration in the 46-dimensional observation space. SAC is therefore selected as the training algorithm.

Soft Actor-Critic (SAC) is an off-policy actor-critic algorithm that augments the standard reinforcement learning objective with an entropy bonus, which encourages exploration while retaining exploitation. Its off-policy nature permits reuse of previously collected transitions, resulting in substantially better sample efficiency than on-policy alternatives. Two independent but structurally identical neural networks parameterize the action-value function and together form the critic module. Taking the minimum of their two estimates at each update step suppresses the overestimation bias that commonly arises in value-based learning. A separate target network, whose weights lag behind the critic through soft averaging, provides stable regression targets during critic updates. The actor is a stochastic policy network from which actions are drawn via the reparameterization trick, enabling gradient-based optimization of the policy parameters. Training proceeds by alternating critic and actor updates until convergence. Because the entropy term appears in the objective functions of both the critic and the actor, the resulting policy is jointly optimized for cumulative reward and stochastic behavior, a property that is particularly useful in the complex parafoil control task considered here.

The objective function of the action-value function is defined as:

15 $$\begin{aligned} J_Q (\theta _i) = \mathbb {E}_{(s_t,a_t,r_t,s_{t+1}) \sim R} \Bigg [ \frac{1}{2} \Bigg ( Q_{\theta _i} (s_t, a_t) - \bigg ( r_t + \gamma \Big ( \min \limits _{j=1,2} Q_{\bar{\theta }_j} (s_{t+1}, a_{t+1}) - \alpha \log \pi (a_{t+1} \mid s_{t+1}) \Big ) \bigg ) \Bigg )^2 \Bigg ] \end{aligned}$$

Where: $Q_{\theta _i}$ represents the two action-value functions of the critic, with $\theta _i$ being the parameters of the corresponding value networks ($i = 1,2$); $Q_{\bar{\theta }_j}$ represents the two target value functions, with $\bar{\theta }_j$ being the parameters of the two value functions ($j = 1,2$); *R* is an experience replay buffer, and $\gamma$ is the discount factor.

The objective function of the policy network is defined as:

16 $$\begin{aligned} J_{\pi } (\phi ) = \mathbb {E}_{s_t \sim R, \epsilon _t \sim \mathcal {N}} \left[ \alpha \log \Big ( \pi _{\phi } ( f_{\phi } (\epsilon _t; s_t) \mid s_t ) \Big ) - \min \limits _{j=1,2} Q_{\bar{\theta }_j} \Big (s_t, f_{\phi } (\epsilon _t; s_t) \Big ) \right] \end{aligned}$$

Where: $\pi _{\phi } (a_t \mid s_t)$ represents the policy’s probability distribution, parameterized as a Gaussian distribution, which is used to compute the probability of taking action $a_t$ under state $s_t$; $f_{\phi } (\epsilon _t; s_t)$ is an action generation function based on the reparameterization trick, mapping state $s_t$ and noise $\epsilon _t$ to the actual action $a_t$, thereby enabling differentiable sampling; $\epsilon _t$ is noise sampled at time *t* from a standard Gaussian distribution, introducing randomness into the action sampling process; $\alpha$ is the temperature parameter that balances the weight between reward and policy entropy during policy optimization.

Instead of treating the temperature parameter as a fixed hyperparameter, SAC adapts $\alpha$ automatically by minimizing the following objective:

17 $$\begin{aligned} J(\alpha ) = \mathbb {E}_{s_t \sim R,\, a_t \sim \pi _t} \left[ -\alpha \log \pi _t (a_t \mid s_t) - \alpha \bar{H} \right] \end{aligned}$$

Here $\bar{H}$ denotes the target entropy, conventionally set to $-\dim (\mathcal {A})$.

**Figure 3:**
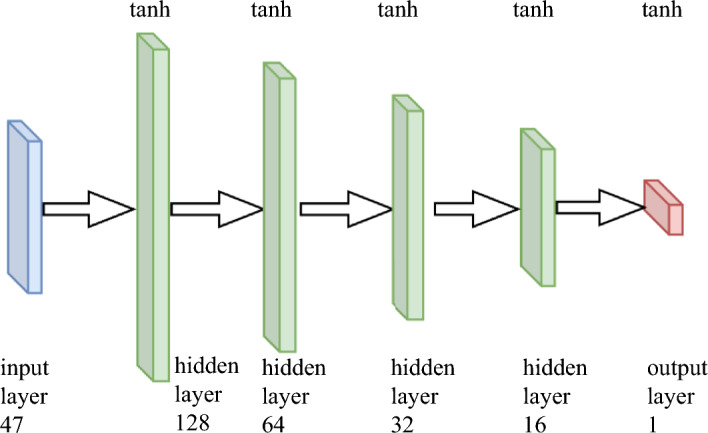
Architecture of the Critic Network.

**Figure 4:**
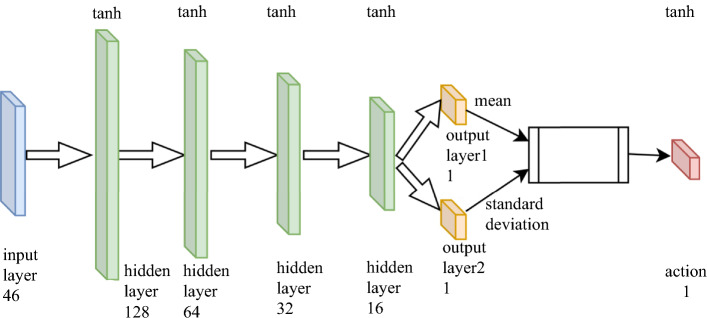
Architecture of the Actor Network.

#### Implementation of SAC4Para

Both the critic and actor are implemented as fully connected neural networks (Figure 3 and 4). The critic receives a 47-dimensional input formed by concatenating the observation state with the action. This input is propagated through four hidden layers of size 128, 64, 32, and 16, each activated by $\tanh$, and a final $\tanh$ unit outputs the scalar action-value estimate. The actor shares the same hidden-layer architecture but takes only the 46-dimensional observation state as input. Its output branches into two independent heads, one producing the mean $\mu$ and the other the standard deviation $\sigma$ of a univariate Gaussian distribution. An action sample is then drawn from $\mathcal {N}(\mu ,\sigma ^{2})$ through the reparameterization trick.

In SAC4Para, training alternates between a data-sampling phase and a network-updating phase until a given stopping criterion is satisfied. During the data-sampling phase, the agent interacts with the parafoil simulation environment and stores every observed transition $(\boldsymbol{s_t}, \boldsymbol{a_t}, r_t, \boldsymbol{s_{t+1}})$ in the replay buffer. In each subsequent update phase, *K* gradient steps are performed using a mini-batch of *m* transitions drawn at random from the buffer; the critic parameters, actor parameters, and temperature $\alpha$ are updated in that order. After training, the resulting policy serves as the parafoil trajectory tracking controller (denoted the SAC4Para controller). Algorithm 4 summarizes the complete procedure.

**Algorithm 4:**
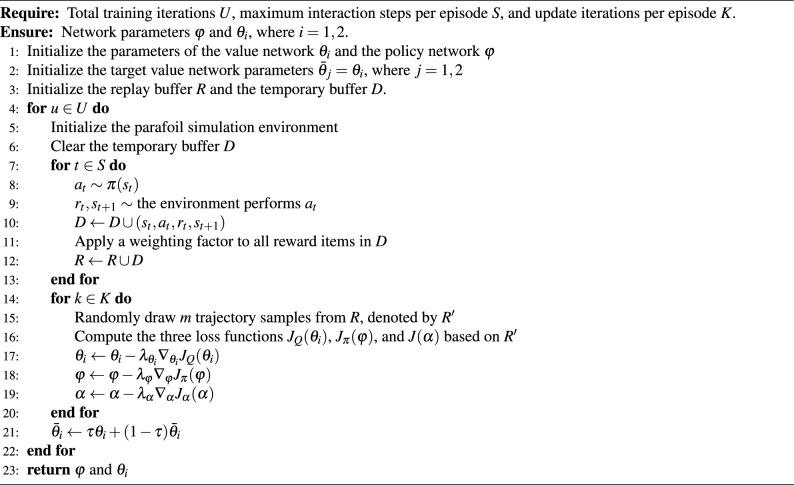
SAC4Para

## Simulation results and analysis

In this section, simulation experiments are conducted to evaluate the BTSG-SAC framework in terms of three evaluation metrics: horizontal distance deviation, heading angle deviation, and control action magnitude. Horizontal distance deviation is defined as the Euclidean distance from the parafoil system’s current position to the point on the planned trajectory at the same altitude, which measures the lateral deviation from the reference path. Heading angle deviation is the absolute angular difference between the horizontal parafoil velocity and the planned trajectory tangent, evaluated at the same altitude.

As in training, the parafoil trajectory tracking task is treated as an episodic task during evaluation. The maximum time step *S* is set to 300, corresponding to a flight at approximately 1000 meters altitude. Over each episode, the mean, standard deviation, and maximum of horizontal distance deviation (DDM, DDSTD, DDMAX), heading angle deviation (HDM, HDSTD, HDMAX), and absolute control value (AAM, AASTD, AAMAX) are recorded. Smaller distance and heading-angle deviations indicate better tracking accuracy, while a small standard deviation of the absolute control value reflects smooth actuation and overall system stability.

Three controllers are trained with the BTSG-SAC framework on the task sets defined in Sect. Benchmark task set generation algorithm: SAC-H on HTT, SAC-S on STT, and SAC-C on CTT. The SAC-H controller is first analyzed to quantify trajectory tracking accuracy. A cross-controller comparison then highlights how HTT contributes to robust generalization. Ablation experiments further analyze the influence of individual state components on controller performance.

All experiments ran on a single PC with an i7-11700K CPU and an NVIDIA GeForce RTX 3060 GPU under Ubuntu 20.04 LTS. Training a controller such as SAC-H took approximately 30 hours, dominated by agent–environment interaction sampling. Training hyperparameters are listed in Appendix E.

### Performance evaluation

PID and PPO are chosen as baselines following He et al.^37^, the closest prior work, providing a classical-control versus DRL comparison. Other controllers in Sect. Introduction, such as LADRC^5,16^ and SMC^6^, were developed for powered parafoil platforms with platform-specific gain models—e.g., Zheng et al.^5,16^ model yaw angular acceleration as linear in the control input, whereas Leeman et al.^39^ adopt a yaw-rate-level formulation closer to the present 9-DOF dynamics. Because the gain parameter has a different physical meaning under each formulation, porting these controllers here would require dedicated re-derivation and tuning, which we leave for future work.

The SAC-H controller is compared against PID and PPO baselines on HTE, which has the same distribution as the training set HTT. The PID baseline adopts the heading angle-based design of^37^. Its gains were initialized at $[k_p = 0.72, k_i = 0.002, k_d = 0.90]$^37^ and subsequently tuned to $[k_p = 0.78, k_i = 0.001, k_d = 1.70]$ through iterative trials in the parafoil simulation environment. The PPO baseline follows^37^ and was trained on HTT under identical conditions.

#### Performance evaluation in noise-free conditions

**Figure 5:**
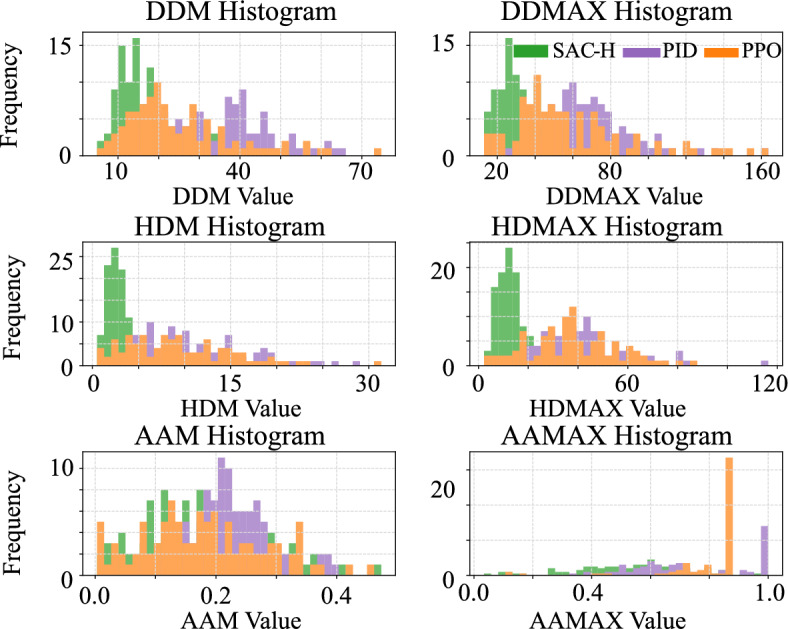
Histograms of evaluation metrics for SAC-H, PPO and PID controllers on the HTE.

Flight experiments were performed on 100 tasks from the HTE, with each task tested once using the SAC-H, PPO, and PID controllers. For each flight task, nine evaluation metrics were calculated. Statistical analyses were then performed on the 100 sets of evaluation metrics. The averages of the mean and standard deviation metrics were computed, while the maxima of the maximum value metrics were determined. The statistical results and distribution characteristics of these metrics are presented in Table 2 and Figure 5.

**Table 2:** Statistical analysis of evaluation metrics for SAC-H, PPO, and PID controllers on the HTE.

Controller	DDM (mean)	DDSTD (mean)	DDMAX (max)	HDM (mean)	HDSTD (mean)	HDMAX (max)	AAM (mean)	AASTD (mean)	AAMAX (max)
SAC-H	**16.919**	** 9.053**	**121.874**	**2.808**	**2.660**	**40.153**	**0.174**	**0.137**	0.961
PPO	25.337	14.317	162.292	9.387	8.540	86.643	0.179	0.186	**0.875**
PID	34.339	17.426	163.568	11.508	10.186	116.361	0.222	0.173	1.000

Table 2 collects the full set of evaluation metrics for the three controllers. In horizontal distance deviation, SAC-H achieves a DDM of 16.919 with a DDSTD of 9.053, roughly 33% and 51% lower than the corresponding PPO and PID values, respectively. The histogram in Figure 5 confirms this trend: SAC-H deviations cluster below 20 m, whereas PID counts spread well into the high-deviation tail. The worst-case DDMAX of SAC-H (121.874) stays well below the 200 m safety threshold and is roughly 40 m smaller than the PPO and PID peaks (162.292 and 163.568).

The advantage is even more pronounced for heading angle deviation. SAC-H maintains an HDM of only 2.808$^\circ$ (HDSTD 2.660$^\circ$), less than one-third of the PPO figure (9.387$^\circ$) and one-quarter of PID (11.508$^\circ$). The corresponding HDMAX values—40.153$^\circ$ for SAC-H versus 86.643$^\circ$ (PPO) and 116.361$^\circ$ (PID)—further indicate tighter directional control for the SAC-based policy. Figure 5 shows that both baseline controllers exhibit a long tail toward large heading errors, which is largely absent from the SAC-H distribution.

Control effort, by contrast, differs only marginally among the three controllers (AAM: 0.174 for SAC-H, 0.179 for PPO, 0.222 for PID), suggesting that the tracking improvements of SAC-H are not achieved at the cost of aggressive actuation. A practically relevant distinction also appears in the tail of the distribution: actions above 0.8 are generally regarded as unsafe for the parafoil system, and such events occur less frequently under SAC-H than under either baseline (Figure 5).

These results can be interpreted in light of the reward weights: the factors $\alpha = 0.5$, $\beta = 0.4$, and $\gamma = 0.1$ penalize heading angle deviation, distance deviation, and control action smoothness, respectively, placing 90% of the total penalty on distance and heading accuracy. It is therefore natural that SAC-H shows the largest gains in DDM and HDM, while the comparatively modest gap in AAM reflects the smaller weight assigned to actuation cost.

**Table 3:** 99% Confidence Intervals of DDM, HDM, and AAM for SAC-H, PPO, and PID Controllers over 100 Simulation Tasks.

Controller	DDM (99% CI)	HDM (99% CI)	AAM (99% CI)
SAC-H	**16.919 ± 0.204**	**2.808 ± 0.047**	**0.174 ± 0.003**
PPO	25.337 ± 0.319	9.387 ± 0.166	0.179 ± 0.003
PID	34.339 ± 0.359	11.508 ± 0.183	0.222 ± 0.003

To assess statistical significance, 100 simulation episodes per controller are used to compute 99% confidence intervals for DDM, HDM, and AAM. Table 3 reports these intervals. All confidence intervals are narrow; in particular, the SAC-H intervals do not overlap with those of PPO or PID for either DDM or HDM, indicating that the observed performance gaps are statistically meaningful.

**Figure 6:**
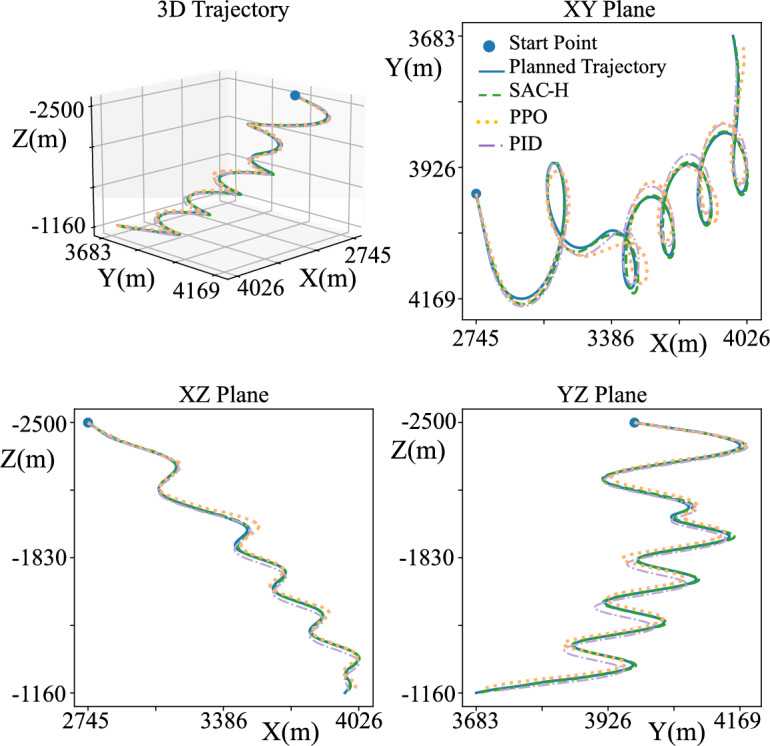
Trajectory tracking performance for the SAC-H, PPO and PID controllers in a random task.

**Figure 7:**
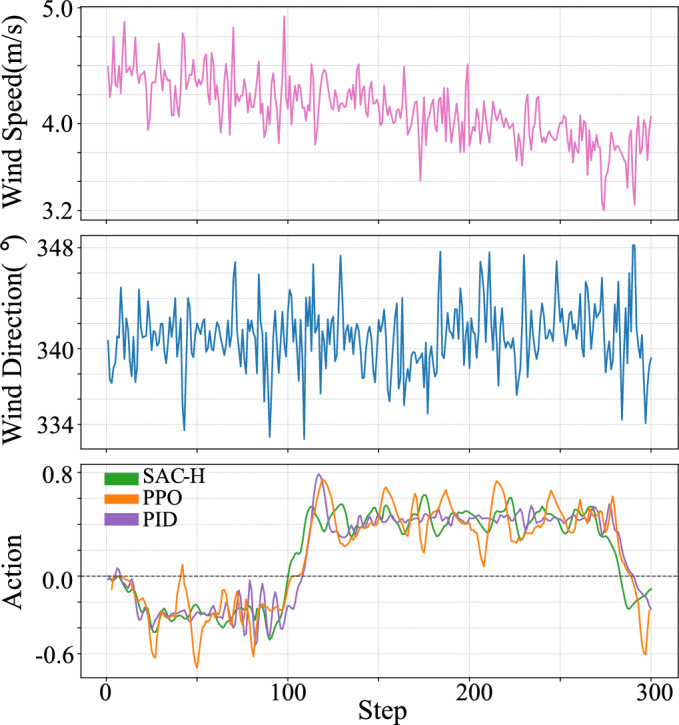
Wind field and action information for the SAC-H, PPO and PID controllers under a randomly selected task.

A randomly drawn episode is then examined to illustrate controller behavior in detail. Figure 6 plots the 3-D trajectory together with its XY, XZ, and YZ projections. SAC-H tracks the reference path closely throughout, with the largest differences from the baselines visible in curved and turning segments. The corresponding wind profile and control histories appear in Figure 7. Wind speed varies between 3.2 and 4.9 m/s with frequent gusts, while the direction oscillates within a 15$^\circ$ band around 340$^\circ$, conditions representative of a turbulent low-altitude environment. Despite these disturbances, SAC-H maintains comparatively smooth actuation; the PPO and PID traces, by contrast, exhibit higher-frequency oscillations, suggesting greater sensitivity to wind variations.

#### Performance evaluation in noisy conditions

To assess the robustness and generalization of the SAC-H controller under noisy conditions, noise was introduced into key sensor-level observations to emulate measurement inaccuracies encountered during flight. Compared with directly perturbing the observation state, introducing noise at the sensor level better represents the system perception process and supports a more representative evaluation setting.

Four types of sensor noise are designed in this study, as detailed below:

**(1) GPS Position Noise:** The WN + FN model^40^, which combines white noise (WN) and flicker noise (FN), is adopted as a representative modeling approach in the field of navigation. The white noise is modeled as zero-mean Gaussian noise with a standard deviation of 5.0, which falls within the typical range for civilian-grade GPS systems [1.5, 5.0]. The spectral decay factor of the flicker noise is set to 1.0, which lies within the commonly used range of 1.0 to 1.5. This noise affects the observation state subvectors PTV, PTA, DTV, DTA, FTC, and FTV, comprising a total of 38 scalar elements.

**(2) GPS Velocity Noise:** Zero-mean Gaussian white noise is applied with a standard deviation of 0.3, which falls within the typical range for civilian-grade GPS systems [0.1, 0.3], and primarily affects the CV subvector, consisting of 3 scalar elements.

**(3) Control Motor State Noise:** Zero-mean Gaussian noise with a standard deviation of 0.1 is introduced to simulate minor measurement errors in control motor sensors, affecting the ACO subvector and comprising 2 scalar elements.

**(4) Airspeed Tube Sensor Noise:** Zero-mean Gaussian noise with a standard deviation of 2.0—corresponding to 10% of the maximum wind speed (20 m/s, as defined in Appendix B)—is applied to the WV subvector, consisting of 3 scalar elements.

The above noise designs are applied not only to the SAC-H controller, but also to the PPO and PID controllers, introducing consistent perturbations to their observation inputs to ensure a fair comparison.

On the HTE, the same evaluation as in Sect. Performance evaluation in noise-free conditions was performed for the SAC-H, PPO, and PID controllers. Across the same 100 flight tasks, the SAC-H controller failed in 2 tasks due to distance deviation exceeding the maximum distance threshold, PPO failed in 23 tasks, and the PID controller failed to achieve effective tracking in all tasks. Table 4 presents the performance statistics of the SAC-H and PPO controllers based on successfully completed tasks, with 98 valid task samples for SAC-H and 77 for PPO.

**Table 4:** Statistical evaluation results of SAC-H and PPO controllers under sensor noise on the HTE (based on successfully completed tasks).

Controller	DDM (mean)	DDSTD (mean)	DDMAX (max)	HDM (mean)	HDSTD (mean)	HDMAX (max)	AAM (mean)	AASTD (mean)	AAMAX (max)
SAC-H	**27.334**	**13.778**	**104.093**	**4.837**	**4.371**	**46.094**	**0.189**	**0.153**	**0.964**
PPO	47.957	28.424	196.990	12.118	10.440	96.465	0.298	0.283	1.000

The results show that SAC-H maintains stable performance under noisy conditions. Compared with noise-free conditions (Table 3), the mean distance deviation of SAC-H increases from 16.919 to 27.334 (61.5%), whereas PPO exhibits a larger increase from 25.337 to 47.957 (89.3%). For heading angle deviation, the mean value of SAC-H increases from 2.808 to 4.837, while that of PPO rises from 9.387 to 12.118. In addition, SAC-H achieves a success rate of 98%, compared with 77% for PPO, whereas PID does not achieve successful tracking under sensor noise. These observations are consistent with the robustness of the BTSG-SAC approach under sensor uncertainty.

### The role of HTT

This section analyzes the performance of three controllers across different evaluation sets. Performance on evaluation sets drawn from the same distribution as the training data reflects fitting ability, whereas performance on sets with differing distributions reflects generalization capability.

**Figure 8:**
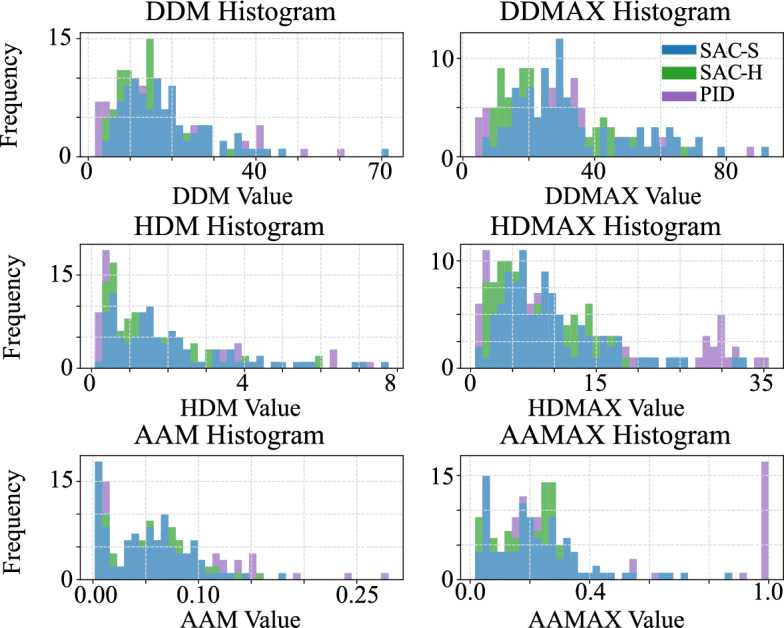
Histograms of evaluation metrics for SAC-S, SAC-H, and PID controllers on the STE.

For STE, 100 flight episodes are run per controller (SAC-S, SAC-H, and PID). Results are collected in Table 5 and Figure 8. SAC-H outperforms both SAC-S and PID on most metrics. SAC-S was trained on STT, whose distribution matches STE, yet it still falls short of SAC-H—an indication that simple homogeneous task data limit high-precision tracking even under in-distribution conditions. The fact that PID (DDM: 15.610) surpasses SAC-S (DDM: 18.301) on STE further indicates that STT alone is insufficient for producing a competitive learned controller.

**Table 5:** Statistical analysis of evaluation metrics for SAC-S, SAC-H, and PID controllers on the STE.

Controller	DDM (mean)	DDSTD (mean)	DDMAX (max)	HDM (mean)	HDSTD (mean)	HDMAX (max)	AAM (mean)	AASTD (mean)	AAMAX (max)
SAC-S	18.301	7.872	92.852	1.982	2.000	32.187	0.052	0.047	0.855
SAC-H	**14.777**	6.513	**70.711**	**1.621**	**1.592**	**18.269**	**0.051**	**0.043**	**0.391**
PID	15.610	**6.194**	86.981	1.739	2.041	35.594	0.063	0.067	1.000

**Figure 9:**
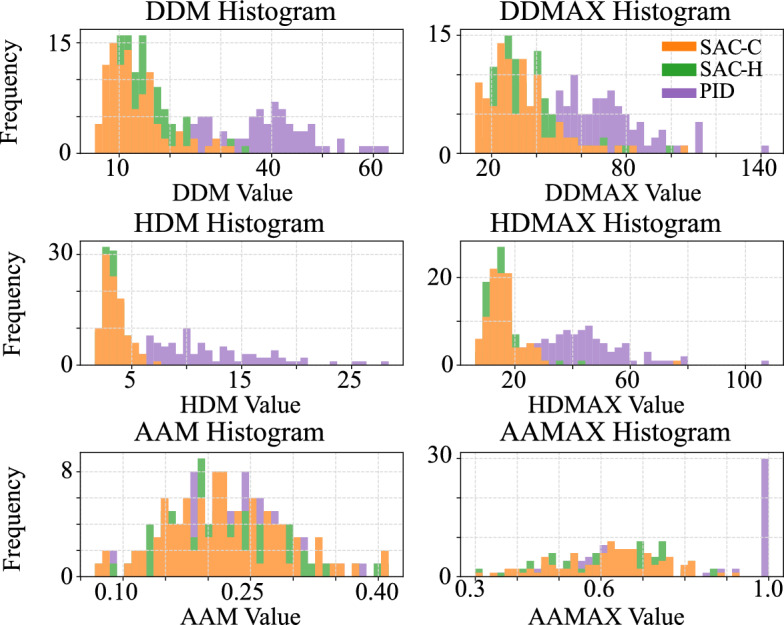
Histograms of evaluation metrics for SAC-C, SAC-H, and PID controllers on the CTE.

The same protocol is repeated on CTE (100 episodes each for SAC-C, SAC-H, and PID). Table 6 and Figure 9 report the results. Both SAC-based controllers dominate PID across every metric, confirming that the learned policies exploit environmental observations more effectively than the fixed-gain baseline in complex scenarios. The SAC-C controller attains a DDM of 13.108 on CTE, the lowest among all three controllers, reflecting strong in-distribution fitting. More notably, the DDM gap between SAC-C and SAC-H on CTE is markedly smaller than the SAC-S–to–SAC-H gap observed on STE. This narrowing suggests that richer training distributions such as CTT extract near-optimal control strategies from the learning algorithm, whereas homogeneous sets like STT leave substantial room for improvement.

**Table 6:** Statistical analysis of evaluation metrics for SAC-C, SAC-H, and PID controllers on the CTE.

Controller	DDM (mean)	DDSTD (mean)	DDMAX (max)	HDM (mean)	HDSTD (mean)	HDMAX (max)	AAM (mean)	AASTD (mean)	AAMAX (max)
SAC-C	**13.108**	**7.903**	105.365	3.427	3.243	75.887	0.218	0.161	0.924
SAC-H	14.846	8.440	**98.090**	**3.282**	**3.060**	**42.577**	**0.216**	**0.159**	**0.875**
PID	33.530	16.882	143.456	11.402	10.227	108.055	0.223	0.177	1.000

**Figure 10:**
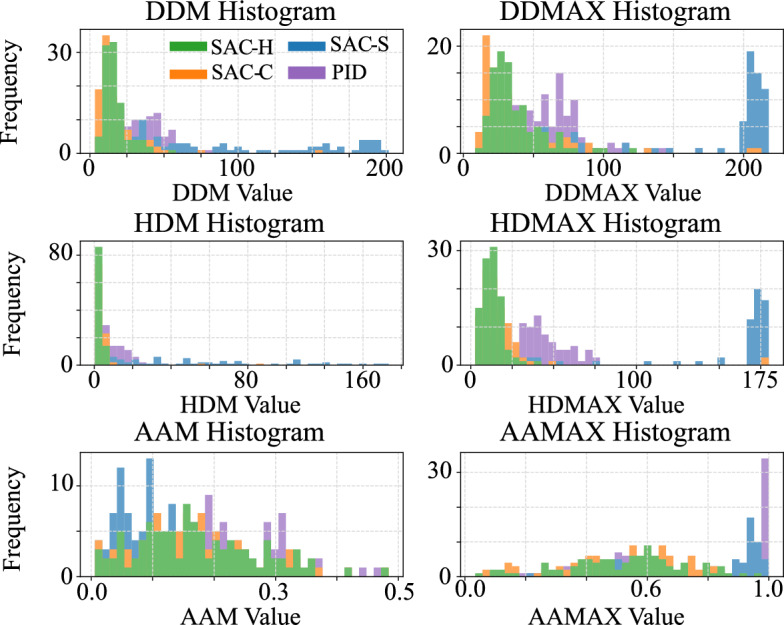
Histograms of evaluation metrics for SAC-H, SAC-S, SAC-C, and PID controllers on the HTE.

All four controllers (SAC-S, SAC-C, SAC-H, PID) are evaluated on HTE; results appear in Table 7 and Figure 10. SAC-H attains the lowest DDM, HDM, and AASTD among the four controllers. SAC-S exhibits severe degradation on HTE: 52 out of 100 episodes are terminated for exceeding the maximum safety distance, indicating poor cross-distribution generalization for a policy trained solely on simple trajectories. The SAC-C controller is close to SAC-H on most metrics yet still incurs two safety-distance violations in 100 episodes, suggesting that CTT-trained policies do not fully transfer to the broader HTE distribution. SAC-H, trained on the more heterogeneous HTT, completes all 100 episodes without violation, indicating that task-set diversity and balanced complexity during training play an important role in achieving robust generalization within the BTSG task family.

**Table 7:** Statistical analysis of evaluation metrics for SAC-S, SAC-C, SAC-H, and PID controllers on the HTE.

Controller	DDM (mean)	DDSTD (mean)	DDMAX (max)	HDM (mean)	HDSTD (mean)	HDMAX (max)	AAM (mean)	AASTD (mean)	AAMAX (max)
SAC-H	**16.919**	**9.053**	**121.874**	**2.808**	**2.660**	**40.153**	0.174	**0.137**	0.961
SAC-S	79.016	43.945	217.858	36.595	24.697	179.925	**0.107**	0.145	0.985
SAC-C	17.797	10.276	211.263	4.524	4.217	177.737	0.174	0.138	**0.842**
PID	34.200	15.432	167.605	9.530	8.948	107.361	0.190	0.161	1.000

Taken together, the three evaluation sets show a consistent pattern linked to training-set design. STT, the simplest set, yields the weakest controller: SAC-S lags behind SAC-H even on in-distribution STE and fails in 52 of 100 HTE episodes. CTT substantially improves performance—SAC-C fits CTE well and nearly matches SAC-H on HTE—yet the two safety-distance violations on HTE indicate that increased complexity alone is not sufficient; coverage across difficulty levels is also important. HTT, which balances easy, moderate, and hard trajectories, produces the only controller (SAC-H) that leads on every metric across all three evaluation sets and completes all 300 episodes without a safety violation. These results indicate that benchmark task-set construction is a core factor in DRL-based controller development. The BTSG algorithm (Algorithm 3) formalizes this construction process and provides a systematic alternative to ad-hoc trajectory sampling. Since all evaluation task sets are produced by the same BTSG algorithm under different parameter configurations, the generalization observed here is cross-distribution within a single procedural generation paradigm; testing against fundamentally different trajectory generation methods remains for future work.

### Ablation study

Section The role of HTT already ablates the effect of training task set design by comparing controllers trained on STT, CTT, and HTT; this section complements that analysis by examining how each observation state component contributes to tracking performance. Ablation experiments are conducted to isolate the contribution of each observation-state component to controller performance. The nine sub-vectors of the 46-dimensional observation state (Table 1) are grouped into five sub-states: $\boldsymbol{s_1}$ (velocity) $=$ {CV}; $\boldsymbol{s_2}$ (target point) $=$ {PTV, PTA, DTV, DTA}; $\boldsymbol{s_3}$ (forward trajectory) $=$ {FTC, FTV}; $\boldsymbol{s_4}$ (actuator output) $=$ {ACO}; and $\boldsymbol{s_5}$ (wind field) $=$ {WV}.

Inspired by the dropout paradigm, each sub-state is ablated in turn by zeroing its entries in the observation vector during both training and evaluation, while the network architecture and all other components remain unchanged. All ablation controllers are trained on HTT and evaluated on HTE.

**Figure 11:**
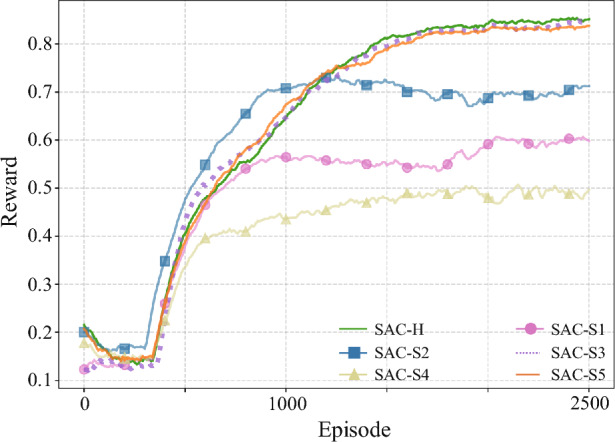
The effects of different sub-states on the training process (SAC-Si denotes the controller trained without sub-state Si, where i =1, 2, ..., 5).

Training curves for each ablation variant are plotted in Figure 11. The full-state baseline (SAC-H) converges within roughly 2000 episodes and plateaus at the highest reward level. Dropping $\boldsymbol{s_1}$, $\boldsymbol{s_2}$, or $\boldsymbol{s_4}$ prevents the reward from improving beyond its initial range, indicating that velocity, target-point, and actuator-output information are essential for the policy to acquire effective control behavior. Without $\boldsymbol{s_3}$ or $\boldsymbol{s_5}$, the agent still reaches a stable plateau, but at a noticeably lower reward level than in the full-state case. Thus, forward-trajectory and wind-field information act mainly to enhance performance rather than to determine the basic convergence of training.

**Table 8:** Evaluation metrics for SAC-H, SAC-S3, and SAC-S5 controllers on the HTE.

Controller	DDM (mean)	DDSTD (mean)	DDMAX (max)	HDM (mean)	HDSTD (mean)	HDMAX (max)	AAM (mean)	AASTD (mean)	AAMAX (max)
SAC-H	**16.919**	**9.053**	**121.874**	**2.808**	**2.660**	**40.153**	**0.174**	**0.137**	**0.961**
SAC-S3	25.201	15.969	214.630	7.105	6.368	177.803	0.178	0.150	0.981
SAC-S5	19.981	11.072	123.778	4.318	4.173	48.571	0.179	0.147	0.953

The three successfully converged controllers—SAC-H, SAC-S3 (trained without $\boldsymbol{s_3}$), and SAC-S5 (trained without $\boldsymbol{s_5}$)—are compared on HTE in Table 8. SAC-H achieves the lowest means and standard deviations for DDM, HDM, and AAM, together with markedly smaller DDMAX and HDMAX. SAC-S3 shows the largest degradation: it fails in 8 out of 100 episodes and exhibits substantially higher deviations on all metrics, indicating that forward-trajectory information is critical for accurate and safe tracking. SAC-S5 lies between the two: no episodes fail, but DDM and HDM increase relative to SAC-H, so wind-field information, while less influential than forward-trajectory data, still provides a measurable benefit to tracking precision.

The inconsistency between the insensitivity observed during training and the performance drop during evaluation may be attributed to two factors. First, for the forward trajectory sub-state, $\boldsymbol{s}_3$ is constructed with a fixed number of points and a fixed sampling distance (see Appendix C), which may not be the form that best adapts to the task. Second, for the wind field sub-state, $\boldsymbol{s}_5$ may exhibit relatively strong stochastic fluctuations (see Figure 11), so the agent may down-weight this input during training in order to obtain a more stable policy.

In summary, the ablation results divide the five sub-states into two functional groups. Sub-states $\boldsymbol{s_1}$, $\boldsymbol{s_2}$, and $\boldsymbol{s_4}$ form a critical core: removing any one of them prevents the policy from converging. Sub-states $\boldsymbol{s_3}$ and $\boldsymbol{s_5}$ are supplementary; training still converges without them, but evaluation-time tracking accuracy degrades, particularly when $\boldsymbol{s_3}$ is absent. These observations suggest that forward-trajectory and wind-field information are beneficial but that their current parameterizations may not yet be optimal, leaving room for further refinement of these sub-states.

## Conclusions

This study addresses parafoil trajectory tracking through three interconnected contributions. First, the Benchmark Task Set Generation (BTSG) algorithm constructs task sets whose complexity is both diverse and proportionally balanced, providing a systematic basis for controller training and for assessing generalization, including cross-distribution generalization within the BTSG-defined task family. Second, the trajectory tracking problem is formulated as a Markov Decision Process over a 46-dimensional observation state with a dense reward signal, facilitating policy optimization with rich decision-relevant information. Third, these elements are integrated in the BTSG-SAC framework, which couples BTSG-generated task sets with a Soft Actor-Critic-based training loop to obtain the parafoil trajectory tracking controller.

Simulation results show that the SAC-H controller trained under BTSG-SAC outperforms both PID and PPO baselines across all tested conditions. In noise-free episodes, SAC-H achieves a DDM of 16.919 m on HTE, versus 25.337 m (PPO) and 34.339 m (PID). When sensor noise is introduced, SAC-H maintains a 98% completion rate, whereas PPO drops to 77% and PID fails to complete any episode. Across the 300 cross-task evaluation episodes, SAC-H incurs zero failures when trained on HTT, indicating stable generalization across task distributions.

These findings support the use of deep reinforcement learning for trajectory tracking in underactuated parafoil systems. The BTSG algorithm provides a structured way to construct training and evaluation task sets, and the BTSG-SAC training pipeline offers a practical framework for applying reinforcement learning in control-oriented applications where systematic task-set design and robust policy learning are both required.

This study has several limitations. The 9-DOF simulator adopts three common modeling assumptions—fixed canopy shape, rigid suspension lines, and payload-drag-only aerodynamics^6,17,37^—and the actuator model from He et al.^37^ does not account for communication latency or response lag. All experiments use a single parafoil configuration, so robustness to model mismatch, actuator delays, and canopy parameter variation remains unverified, and no sim-to-real transfer has been attempted. Formal Lyapunov-type stability analysis for deep-RL policies remains an open challenge^41,42^; the convergence, noise-robustness, and cross-task results reported in Sects. Performance evaluation–Ablation study offer empirical evidence but not a theoretical guarantee.

Future work will focus on three main directions. First, the ablation results indicate that the current parameterizations of the forward-trajectory and wind-field sub-states ($\boldsymbol{s_3}$ and $\boldsymbol{s_5}$) are less effective than the core sub-states; refining their representations in the observation space is expected to further improve tracking accuracy and robustness. Second, continual learning and cross-platform transfer are of interest, in order to enhance long-term adaptability and support deployment across parafoil systems with different canopy geometries and payload configurations. Third, flight-test validation on a physical parafoil platform is needed to assess the practical applicability and robustness of the proposed approach under real operational conditions, particularly regarding the sim-to-real gap arising from model mismatch, actuator delays, and canopy parameter variation identified above.

## Figures and Tables

**Figure 12:**
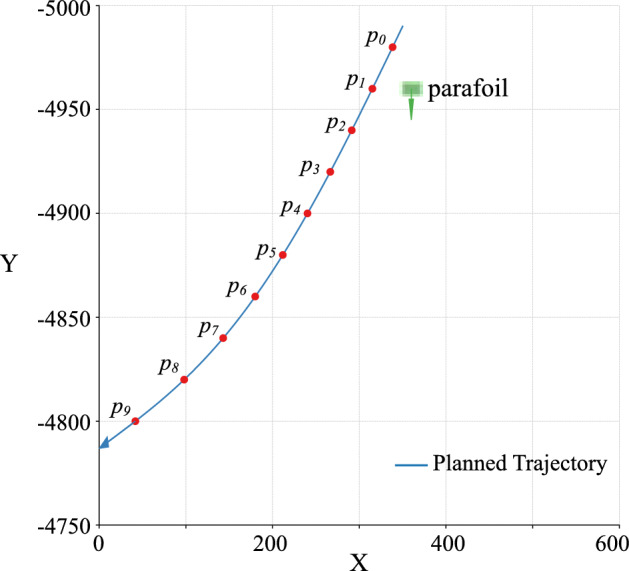
Schematic of Forward Points.

## Data Availability

Data is contained within the article or supplementary material.

## Appendix A: Supplementary notes for 9-DOF model of parafoil system

This appendix provides the expanded right-hand-side terms of Eq. (6), and the symbol definitions related to Eqs. (3)–(6) in the nine-DOF dynamic model.

The vectors appearing on the right-hand side of Eq. (6) are given by:

18 $$\begin{aligned} \begin{bmatrix} \boldsymbol{B_1} \\ \boldsymbol{B_2} \\ \boldsymbol{B_3} \\ \boldsymbol{B_4} \end{bmatrix} = \begin{bmatrix} \boldsymbol{F_{ab}} + \boldsymbol{F_{gb}} - \boldsymbol{M_b \tilde{\boldsymbol{\omega }}_b \tilde{\boldsymbol{\omega }}_b R_{cb}} \\ \boldsymbol{F_{ap}} + \boldsymbol{F_{gp}} - (\boldsymbol{M_p} + \boldsymbol{M_a}) \boldsymbol{\tilde{\omega }_p \tilde{\boldsymbol{\omega }}_p R_{cp}} \\ \boldsymbol{M_b^{A}} - \boldsymbol{\tilde{\omega }_b I_b \omega _b} \\ \boldsymbol{M_p^{A}} - \boldsymbol{\tilde{\omega }_p (I_p + I_a) \omega _p} \end{bmatrix} \end{aligned}$$

In Eqs. (3)–(6), $t_* = \tan (*)$; $\boldsymbol{X_c}$ represents the translational displacement of the connection point $O_c$, $\boldsymbol{V_c}$ represents the translational velocity of the connection point. $$\boldsymbol{\omega _b} = \begin{bmatrix} p_b&q_b&r_b \end{bmatrix}^T$$ is the angular velocity of the payload in the payload coordinate system, while $$\boldsymbol{\omega _p} = \begin{bmatrix} p_p&q_p&r_p \end{bmatrix}^T$$ is the angular velocity of the canopy in the canopy coordinate system. $\boldsymbol{M_b}$ denotes the mass matrix of the payload, $\boldsymbol{M_p}$ is the actual mass matrix of the canopy, and $\boldsymbol{M_a}$ is the additional mass matrix of the canopy. $\boldsymbol{R_{cb}}$ represents the distance vector from the connection point $O_c$ to the center of mass of the payload $O_b$, and $\boldsymbol{R_{cp}}$ represents the distance vector from $O_c$ to the center of mass of the canopy $O_p$. $\boldsymbol{I_b}$ is the rotational inertia matrix of the payload, $\boldsymbol{I_p}$ represents the rotational inertia matrix of the canopy, $\boldsymbol{I_a}$ is the rotational inertia matrix of the canopy’s additional mass. $\boldsymbol{F_{ab}}$ represents the aerodynamic force acting on the payload, $\boldsymbol{F_{gb}}$ represents the gravitational force acting on the payload, $\boldsymbol{F_{ap}}$ represents the aerodynamic force acting on the canopy, and $\boldsymbol{F_{gp}}$ represents the gravitational force acting on the canopy. $\boldsymbol{F_c}$ denotes the constraint force vector at the connection point $O_c$, which is an internal force of the system. $\boldsymbol{M_b^{A}}$ represents the aerodynamic moment acting on the payload, and $\boldsymbol{M_p^{A}}$ represents the aerodynamic moment acting on the canopy. Finally, the symbol ’ $\tilde{}$ ’ above a vector indicates its antisymmetric matrix.

## Appendix B: Parameters used for generating task sets

Table 9 lists the parameters used in generating the task sets.

**Table 9:** Parameters for generating task sets.

Task Set Name	$V_{m\_min}$	$V_{m\_max}$	$V_{g\_min}$	$V_{g\_max}$	$\sigma _{r_{min}}$	$\sigma _{r_{max}}$	$a_{min}$	$a_{max}$	$t_{min}$	$t_{max}$
HTT	0.0	2.0	0.0	1.5	0.0	1.0	-0.4	0.6	30	180
HTE	0.0	2.0	0.0	1.5	0.0	1.0	-0.4	0.6	30	180
STT	0.0	1.0	0.0	0.75	0.0	0.5	-0.2	0.2	90	180
STE	0.0	1.0	0.0	0.75	0.0	0.5	-0.2	0.2	90	180
CTT	0.0	2.0	0.0	1.5	0.0	1.0	-0.15	0.6	30	60
CTE	0.0	2.0	0.0	1.5	0.0	1.0	-0.15	0.6	30	60

Note that the values of the four parameters $V_{g\_min}$, $V_{g\_max}$, $\sigma _{r_{min}}$, and $\sigma _{r_{max}}$ in Table 9 correspond to those used during the training or evaluation phases. During the task set generation phase, all four parameters are set to zero, meaning that the wind field consists only of mean wind.

## Appendix C: Physical interpretation of observation state components

The observation state is a 46-dimensional vector consisting of 9 sub-vectors, each with a distinct physical meaning. The details are described below.

**C-point Velocity** (CV) is a 3-dimensional sub-vector that represents the velocity of the parafoil system (C-point) in the ground coordinate system. The airspeed of the parafoil system is 10.45 m/s. Taking wind field effects into account, the minimum value of this vector is defined as $[-20, -20, -20]$, and the maximum value as [20, 20, 20].

**Proximal Target Point Vector** (PTV) is a 2-dimensional sub-vector that represents the vector on the horizontal plane pointing from the C-point to the proximal target point. The proximal target point refers to the point on the planned trajectory at the same altitude as the parafoil. The range of this vector is defined as $[-200, -200]$ to [200, 200].

**Proximal Target Point Angle** (PTA) is a 1-dimensional sub-vector that represents the angle between the C-point velocity vector and the PTV. Its value range is defined as $[-\pi , \pi ]$.

**Distal Target Point Vector** (DTV) is a 2-dimensional sub-vector that represents the vector on the horizontal plane pointing from the C-point to the distal target point. The distal target point refers to the point on the planned trajectory located 20 meters below the current altitude of the parafoil. The range of this vector is defined as $[-200, -200]$ to [200, 200].

**Distal Target Point Angle** (DTA) is a 1-dimensional sub-vector that represents the angle between the C-point velocity vector and the DTV. The range of this angle is defined as $[-\pi , \pi ]$.

The forward trajectory refers to the segment of the planned trajectory that the parafoil system is about to follow, that is, the sub-trajectory between points $p_0$ and $p_9$, as shown in Figure 12. Here, $P = \{p_0, p_1, \dots , p_9\}$ represents the 10 forward points arranged along the Z-axis from top to bottom, with a height difference of 20 meters between adjacent points. The point $p_1$ corresponds to the point on the planned trajectory at the same altitude as the parafoil system. The height difference, set at 1.8 times the minimum sub-trajectory height (100 meters in HTT/HTE), is an empirically determined value that effectively captures variations in the forward trajectory.

**Forward Trajectory Curvature** (FTC) is an 8-dimensional sub-vector that represents the curvatures of the horizontal projection of the forward trajectory at eight points: $p_1, p_2, \dots , p_8$. The curvature is calculated by fitting a local curve using a quadratic polynomial based on three points: $p_{i-1}, p_i, p_{i+1}$ ($i = 1, 2, \dots , 8$). The curvature at point $p_i$ is defined as the curvature of the local curve at that point. The value range of the curvature is $[-1, 1]$.

**Forward Trajectory Vector** (FTV) is a 24-dimensional sub-vector ($8 \times 3$) representing the heading vectors at eight points: $p_1, p_2, \dots , p_8$. The heading vector at point $p_i$ is defined as the vector pointing from $p_i$ to $p_{i+1}$. The range of this vector is $[-100, -100, -100]$ to [100, 100, 100].

**Actuator Control Outputs** (ACO) is a 2-dimensional sub-vector that represents the current output signals of the actuator model in the parafoil system. The range of this vector is defined as [0, 0] to [1, 1].

**Wind Vector** (WV) is a 3-dimensional sub-vector that represents wind field information. The range of this vector is defined as $[-20, -20, -20]$ to [20, 20, 20].

Note: When outputting the observation state, the parafoil simulation system normalizes all dimensions to the range $[-1, 1]$ based on their respective value ranges to accelerate training.

## Appendix D: Calculation methods for individual reward components

In the design of the reward function, the horizontal distance deviation from the planned trajectory, heading angle deviation, and control action smoothness of the parafoil are comprehensively considered and calculated as follows:

**Heading Angle Deviation Term** ($H_t$)): To align the parafoil’s flight direction with the planned trajectory direction, a reward term for heading angle deviation is defined and calculated as follows:

19 $$\begin{aligned} H_t = \exp (-A_t) \end{aligned}$$

where $A_t$ represents the angle between the parafoil’s velocity vector and the planned trajectory vector at time step *t*. The planned trajectory vector is the vector from the target point at time $t-1$ to the target point at time *t*.

**Horizontal Distance Deviation Term** ($D_t$): To minimize the horizontal distance deviation between the parafoil system’s C-point and the planned trajectory, $D_t$ is defined and calculated as follows:

20 $$\begin{aligned} D_t = \frac{200 - d_t}{200} \end{aligned}$$

where $d_t$ represents the horizontal distance between the parafoil system and the corresponding target point at the same altitude in the current time step.

**Control Smoothness Term** ($C_t$): To encourage the controller to generate smooth actions, the reward term $C_t$ is defined. This term is calculated as the difference between the current control action and the previous control action, as follows:

21 $$\begin{aligned} C_t = \frac{2 - |a_t - a_{t-1} |}{2} \end{aligned}$$

where $a_t$ and $a_{t-1}$ represent the control action at the current and previous time steps, respectively.

## Appendix E: Hyperparameters

Table 10 lists the hyperparameters used in the controller training.

**Table 10:** SAC4Para training hyperparameters.

Parameter	Value
Optimizer	Adam
Learning rate (critic)	0.002
Learning rate (actor)	0.001
Discount	0.993
Target smoothing coefficient	0.005
